# Early Genotype-Driven Diagnosis of Hermansky-Pudlak Syndrome Type 4 in a Child With Oculocutaneous Albinism: An Ophthalmic Case Report

**DOI:** 10.7759/cureus.114112

**Published:** 2026-08-07

**Authors:** Dorottya Szabo, Julie Waldispühl-Geigl, Sandra Kamper, Heidemarie Pilch, Barbara Plecko, Kathrin Vollnhofer, Laura Posch-Pertl

**Affiliations:** 1 Department of Ophthalmology, Medical University of Graz, Graz, AUT; 2 Diagnostic and Research Institute of Human Genetics, Medical University of Graz, Graz, AUT; 3 Department of Pediatrics and Adolescent Medicine, Medical University of Graz, Graz, AUT

**Keywords:** hermansky-pudlak syndrome, hermansky-pudlak syndrome type 4, hps, occulocutaneous albinism, syndromic albinism

## Abstract

Hermansky-Pudlak syndrome (HPS) is a rare inherited multisystem condition characterized by oculocutaneous albinism, bleeding diathesis, and subtype-specific systemic complications, including pulmonary fibrosis and granulomatous colitis. Early diagnosis is essential because pulmonary involvement, particularly in HPS type 4, may be life-threatening.

We report a boy who was first referred for ophthalmologic evaluation at seven weeks of age due to generalized hypopigmentation and congenital nystagmus. Examination revealed marked iris translucency, diffuse fundus hypopigmentation with prominent choroidal vasculature, pendular horizontal nystagmus, suspected esotropia, and hyperopia. Genetic testing was initiated at five months of age to determine the underlying cause of the oculocutaneous albinism, including possible syndromic causes. It identified biallelic variants in the HPS4 gene, confirming Hermansky-Pudlak syndrome type 4 at eight months of age. Multidisciplinary evaluation, including hematologic and pulmonary baseline assessments, was subsequently initiated. At the latest follow-up, at two years and five months of age, the patient remained clinically stable without evidence of pulmonary or gastrointestinal complications. Visual rehabilitation and early developmental support were implemented.

This case underscores the critical role of early ophthalmologic recognition of oculocutaneous albinism in establishing the diagnosis of Hermansky-Pudlak syndrome. Timely genetic confirmation enables structured surveillance for pulmonary fibrosis and other systemic complications associated with HPS type 4.

## Introduction

Hermansky-Pudlak syndrome (HPS), first described in 1959, is a rare autosomal recessive multisystem disorder caused by defects in lysosome-related organelles [1,2]. These defects impair melanosome and platelet dense-body formation, resulting in oculocutaneous albinism and platelet storage-pool deficiency [2]. The estimated global prevalence ranges from 1:500,000 to 1:1,000,000, with higher frequencies reported in founder populations, particularly among individuals of Puerto Rican descent [3].

To date, 11 genetically distinct subtypes have been identified, each associated with pathogenic variants in genes involved in intracellular vesicle trafficking [3,4]. All subtypes present with oculocutaneous albinism and bleeding diathesis, while HPS types 1, 2, and 4 are specifically associated with progressive pulmonary fibrosis [3,5]. Additional manifestations may include granulomatous colitis or immunodeficiency, depending on the underlying genetic subtype [3]. Pulmonary involvement represents the leading cause of mortality and typically manifests in early adulthood [5]. HPS type 4 is particularly rare but clinically important because of its association with progressive pulmonary fibrosis and potentially life-threatening systemic complications [3-5].

Ophthalmologic manifestations are universal and include iris and retinal hypopigmentation, congenital nystagmus, foveal hypoplasia, reduced visual acuity, photophobia, strabismus, and refractive errors [3,6-9]. At an early age, HPS may resemble nonsyndromic oculocutaneous albinism because the ocular features overlap and systemic manifestations may not yet be clinically apparent [3,9]. Therefore, recognition of oculocutaneous albinism should prompt consideration of syndromic causes and appropriate genetic evaluation.

An early diagnosis of HPS enables timely systemic assessment, surveillance, and management. This includes recognition of the increased bleeding risk before surgical or invasive procedures and monitoring for subtype-specific complications such as pulmonary fibrosis. Ophthalmologists may therefore play an important role in initiating the diagnostic process and facilitating appropriate multidisciplinary care.

We present a pediatric case in which ophthalmologic recognition of oculocutaneous albinism prompted genetic testing, leading to the early diagnosis of Hermansky-Pudlak syndrome type 4.

## Case presentation

A seven-week-old boy was referred by his primary care ophthalmologist to the Departments of Ophthalmology and Pediatrics for further evaluation of suspected albinism. There was no known family history of albinism, bleeding disorders, or pulmonary disease.

Ophthalmologic findings

The patient exhibited generalized hypopigmentation, including white scalp hair and eyelashes. A pendular horizontal congenital nystagmus of moderate frequency and small-to-moderate amplitude was observed. Ocular alignment was estimated to range from orthotropia to approximately 5-8° of esodeviation, as far as could be assessed in the presence of nystagmus.

Visual function assessment demonstrated stable fixation and the ability to follow targets horizontally and vertically. Confrontation testing suggested intact visual fields. Grating acuity was assessed using LEA GRATINGS® (LEA Test Intl., LLC, Elgin, IL, USA), yielding reliable responses; however, grating acuity at this age cannot be directly translated into optotype-equivalent visual acuity.

Anterior segment examination revealed marked iris hypopigmentation with near-complete translucency. Fundus examination demonstrated diffuse retinal hypopigmentation with prominent visualization of the underlying choroidal vasculature. Cycloplegic retinoscopy revealed high hyperopia with astigmatism (right eye: +9.00 +1.50 × 60°; left eye: +8.00 +2.00 × 130°). Given the patient’s young age, optical coherence tomography could not be reliably performed.

Based on the constellation of findings, oculocutaneous albinism was suspected [6,7,9]. Corrective hyperopic spectacles with transition lenses were prescribed to address refractive error and reduce photophobia.

Systemic evaluation

At this young age, there were no other clinical features typically associated with Hermansky-Pudlak syndrome.

Molecular genetic analysis identified a pathogenic variant in the HPS4 gene (NM_022081.6:c.649C>T, p.(Arg217*)) in a homozygous state (Table 1). Both parents were confirmed to be asymptomatic heterozygous carriers. This finding established the diagnosis of Hermansky-Pudlak syndrome type 4 and is consistent with previously reported mutation spectra [3,4].

**Table 1:** Molecular genetic analysis results. Summary of the identified pathogenic HPS4 variant detected in the patient. Molecular testing demonstrated the homozygous variant NM_022081.6:c.649C>T, p.(Arg217*), consistent with an autosomal recessive diagnosis of Hermansky-Pudlak syndrome type 4 [3,4].

Parameter	Result
Gene	HPS4
Transcript	NM_022081.6
Nucleotide variant	c.649C>T
Protein change	p.(Arg217*)
Zygosity	Homozygous
Inheritance pattern	Autosomal recessive

**Figure 1:**
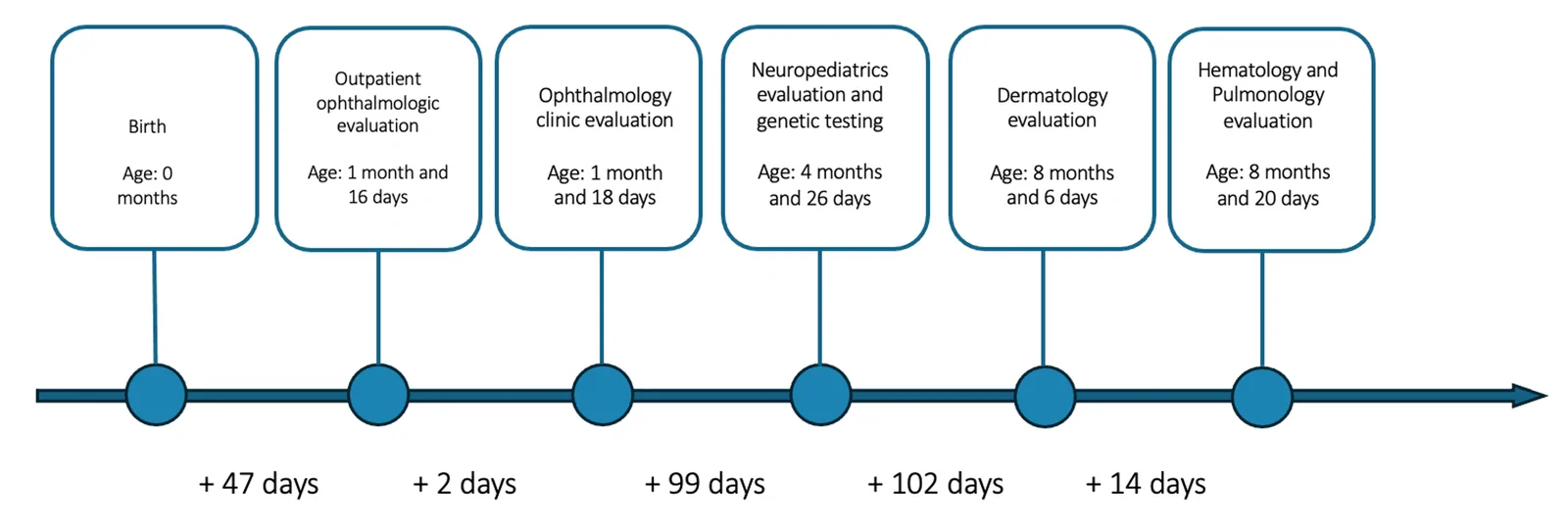
Clinical timeline of diagnosis and multidisciplinary evaluation. Timeline showing the patient’s pathway from birth through ophthalmologic assessment, genetic testing, and subsequent multidisciplinary evaluations.

Baseline multidisciplinary evaluations were initiated, including hematologic assessment of platelet function and pulmonary consultation (Figure 1). Hematologic findings were consistent with a platelet dense-granule defect, characteristic of a delta-storage pool disorder; however, no bleeding events had occurred at the time of reporting. Avoidance of acetylsalicylic acid and nonsteroidal anti-inflammatory drugs was recommended, and desmopressin may be considered before procedures associated with increased bleeding risk.

Pulmonary and gastrointestinal evaluations were unremarkable. Pulmonary function testing is planned from three to four years of age, followed by baseline noncontrast computed tomography of the chest without anesthesia at four to five years of age if the clinical course remains stable. Long-term surveillance for pulmonary fibrosis and potential gastrointestinal involvement was planned in accordance with current recommendations [3,5].

Therapeutic interventions

The patient underwent several months of developmental and vision therapy. A low-vision assessment was performed to optimize visual development, and early intervention services were implemented to support neurodevelopmental outcomes.

At the latest follow-up, the patient remained clinically stable.

## Discussion

Hermansky-Pudlak syndrome is a lysosomal trafficking disorder in which ophthalmologic findings are frequently among the earliest detectable manifestations [2,3,8,9]. In the present case, iris translucency, fundus hypopigmentation, congenital nystagmus, and refractive error prompted genetic testing to investigate the underlying cause of the oculocutaneous albinism, including possible syndromic causes, and led to early molecular confirmation.

At an early age, syndromic and nonsyndromic forms of oculocutaneous albinism may initially present with similar ophthalmologic features, while systemic manifestations may be absent or not yet clinically apparent [3,9]. Therefore, the absence of bleeding or other systemic symptoms at initial presentation does not exclude an underlying syndromic form of albinism [3]. Other differential diagnoses include Chediak-Higashi syndrome and Griscelli syndrome, which may also present with hypopigmentation but differ in their immunologic and hematologic profiles [3]. These disorders involve abnormalities in intracellular trafficking pathways affecting pigmentation and immune function [3].

HPS type 4 is particularly relevant because of its association with progressive pulmonary fibrosis, which significantly impacts long-term prognosis [4,5,10]. Although pulmonary manifestations typically arise in early adulthood, early diagnosis enables anticipatory counseling and structured multidisciplinary surveillance. Such proactive management may facilitate early detection of pulmonary decline and timely referral for advanced therapeutic options, including lung transplantation when indicated [3,5,10].

This case highlights the central role of the ophthalmologist in initiating systemic evaluation for rare multisystem disorders presenting with ocular findings in infancy.

## Conclusions

Oculocutaneous albinism presenting at an early age should prompt consideration of syndromic causes, including Hermansky-Pudlak syndrome. Early molecular confirmation of HPS type 4 enables appropriate multidisciplinary surveillance, facilitating the early detection and timely management of pulmonary and other systemic complications.
